# The Role of Post-Bronchoscopy Sputum Examination in Screening for Active Tuberculosis

**DOI:** 10.3390/tropicalmed8010013

**Published:** 2022-12-26

**Authors:** Gawahir A. Ali, Wael Goravey, Faraj S. Howady, Maisa Ali, Awni Alshurafa, Ahmed M. Abdalhadi, Muhammed Hajmusa, Joanne Daghfal, Abdullatif Al Khal, Muna Al Maslamani, Hussam Al Soub, Ali S. Omrani

**Affiliations:** 1 Division of Infectious Diseases, Department of Medicine, Hamad Medical Corporation, Doha 3050, Qatar; 2 Communicable Diseases Center, Hamad Medical Corporation, Doha 3050, Qatar; 3 Division of Internal Medicine, Department of Medicine, Hamad Medical Corporation, Doha 3050, Qatar; 4 College of Medicine, Qatar University, Doha 2713, Qatar

**Keywords:** tuberculosis, bronchoscopy, post-bronchoscopy smear, diagnosis

## Abstract

Early diagnosis is a fundamental component of global tuberculosis control. The objective of this study was to evaluate the diagnostic yield of post-bronchoscopy sputum (PBS) testing as part of a tuberculosis diagnostic work-up. All new residents in the State of Qatar undergo a tuberculosis (TB) screening program. Those with abnormal chest radiology, negative sputum acid-fast bacilli (AFB) smears, and nucleic acid amplification testing (NAAT) for *M. tuberculosis*, undergo an additional bronchoscopic evaluation for TB. We prospectively enrolled individuals who were going to undergo bronchoscopy to provide two PBS samples for AFB smears and mycobacterial cultures between 18 September 2018 and 12 March 2021. A total of 495 individuals, with a median age of 31 years, were included. The majority of the patients were males (329, 66.5%). The most frequent country of origin was India (131, 26.5%) followed by the Philippines (123, 24.8%). The addition of PBS to bronchoalveolar lavage (BAL) testing allowed microbiological confirmation of tuberculosis in an additional 13 patients (3.9%), resulting in improved sensitivity (from 77.9% to 81.9%), negative predictive value (from 69.2% to 73.2%), and negative likelihood ratio (from 0.22 to 0.18). Where resources are available, the incorporation of routine PBS examination as part of tuberculosis diagnostic work-up can enhance the diagnostic yield.

## 1. Introduction

Globally, tuberculosis (TB) remains a leading cause of morbidity and mortality [1]. Despite the progress made in recent decades in the fight against TB, it remains among the ten leading causes of death from a single infectious agent, infecting approximately 10.6 million and causing an estimated 1.6 million related deaths among people in 2021 [1].

Strategies for global tuberculosis control hinge on early diagnosis, including systematic screening of high-risk populations and effective treatment [2]. However, a considerable delay in TB diagnosis and treatment is often due to the lack of highly sensitive and rapid diagnostic tests [3]. Efforts are required to further improve the diagnostic yield of routine screening for pulmonary tuberculosis [2]. The standard, rapid, and inexpensive method for identifying presumptive active pulmonary tuberculosis cases is sputum smear microscopy and culture with a sensitivity ranges of 30–70% and 80–85%, respectively [4]. However, the sensitivity of smear microscopy is further limited by operator experience and lack of data on susceptibility-guided therapy [5]. In contrast, culture, the gold standard for TB diagnosis, takes up to eight weeks before results can be obtained, delaying early initiation of treatment [6]. The use of sputum nucleic acid amplification testing (NAAT) for *M. tuberculosis*, such as Xpert MTB/RIF, further increases the detection rate of TB with the additional value of rapid assessment of drug susceptibility [7]. Fiberoptic bronchoscopy was further added to the diagnostic yield of sputum smear-negative pulmonary TB with a sensitivity range of 80–93%. However, a proportion of patients with pulmonary TB remains undiagnosed despite repeated sputum and bronchoalveolar lavage (BAL) examination [8]. Thus, the key to achieving a global strategy to control TB is to improve the accuracy of diagnostic assays by identifying optimal methods for pulmonary TB diagnosis in bacteriologically negative patients [5].

There is limited available evidence and considerable uncertainty regarding the role of PBS acid-fast bacilli smear (AFB) and cultures in the diagnosis of pulmonary TB [8,9]. Though the evidence base is limited, current guidelines from the American Thoracic Society (ATS) and Infectious Diseases Society of America (IDSA) suggest performing post-bronchoscopy sputum acid-fast bacilli smear and cultures to increase the diagnostic yield [9]. PBS examination reported up to 8.8% and 7% exclusively positive AFB sputum smear and culture, respectively [9,10,11]. It has been postulated that bronchoscopy may mobilize mycobacteria-laden deep bronchial secretions, resulting in higher diagnostic yields from PBS samples [12]. Therefore, the addition of a post-bronchoscopy sputum smear and culture may identify additional cases of active pulmonary tuberculosis that would have otherwise been missed at the time of screening [8].

In Qatar, where migrant craft and manual workers from countries with a high prevalence of tuberculosis make up the majority of the population, an intensive tuberculosis screening program is in place. The reported TB incidence in Qatar is 40/100,000 population per year, and despite the TB detection rate exceeding 70%, TB remains a common health issue [13].

The aim of this study was to evaluate the diagnostic yield of PBS as part of a tuberculosis diagnostic work-up. Evaluating and comparing the additional microbiological yield of PBS acid-fast bacilli smears and cultures will assist in comprehending and identifying the optimal methods for pulmonary TB diagnosis in bacteriologically negative patients, developing future control and prevention programs, and ultimately improving clinical outcomes.

## 2. Materials and Methods

We performed a prospective study of adult patients who were investigated for pulmonary TB based on radiologic and/or clinical presentations from 18 September 2018 to 12 March 2021 in the Communicable Disease Center (CDC), a tertiary referral center in Doha, Qatar. Qatar is a Gulf country in the Arabian Peninsula with a dynamic and diverse population that has grown substantially over the last two decades. The estimated population in 2009 was 1.8 million, which grew steadily to 2.8 million in 2019. Residency procedures for new arrivals in Qatar, and renewals for certain occupations, include undergoing a chest radiograph-based tuberculosis screening program. Those with abnormal imaging findings are referred for a detailed history and physical examination, tuberculin skin testing or QuantiFERON-TB Gold Plus (Qiagen, Düsseldorf, Germany), expectorated or induced sputum samples for AFB smears, nucleic acid amplification testing (NAAT) for *Mycobacterium tuberculosis*, and mycobacterial cultures. Individuals who cannot produce sputum and those whose expectorated sputum AFB smear and NAAT are negative undergo bronchoscopy and their BAL fluid is sent for AFB smears, NAAT, and culture. We prospectively enrolled all patients aged 18 years and above whose tuberculosis screening included bronchoscopy to collect two PBS samples (1 h apart within 24 h after bronchoscopy and sent for AFB smears and mycobacterial cultures) during the study period. Presumptive TB cases were selected for bronchoscopy at the discretion of the treating physicians responsible for their care with no influence from the investigators implementing this study. All methods were performed in accordance with the relevant guidelines and regulations of the Hamad Medical Corporation Institutional Review Board (MRC) and the Declaration of Helsinki [14].

### 2.1. Study Definition, Collection of the Sputum, and Bronchoscopy BAL Technique

Active tuberculosis was defined as positive NAAT and/or smears and culture for *M. tuberculosis*, or improvement of symptoms and/or radiological abnormalities after between 6 and 8 weeks of anti-tuberculous therapy [2]. The collection of sputum for AFB in the CDC follows the National Reference TB Laboratory, Hamad Medical Corporation (HMC) guidelines, and international standards [15].

For pre-bronchoscopy sputum, first-morning sputum samples were collected on two separate days (two AFB smears, NAAT, and culture for *M. tuberculosis*). For post-bronchoscopy assessment, two AFB smears, at least 1 h apart, and one AFB culture for *M. tuberculosis* were collected. All post-bronchoscopy samples were obtained within 24 h of bronchoscopy. Patients were asked to provide deep, thick mucoid sputum and not saliva (rinsing the mouth with tap water, breathing deeply, and coughing several times). Sputum was expectorated into a dry, sealed, sterile, and labelled container. For patients who were unable to produce sputum, induction of sputum was performed by a trained nurse (using hypertonic 3%–5% saline nebulization). BAL was performed according to the American Thoracic Society (ATS) guidelines [9]. Tracheobronchial anesthesia was induced by intravenous Midazolam/Fentanyl with an upper airway topical 10% Xylocaine spray. A flexible bronchoscope with a minimum internal diameter of 2.0 mm was advanced to the desired affected segment of the lung. Approximately between 100 and 200 mL of normal saline was injected into the affected segments, and at least between 10 and 30% of the infused volume was collected and delivered to the laboratory immediately. For any suspected TB samples, fluorescence and Ziehl-Neelsen (ZN) staining techniques, NAAT for *Mycobacterium tuberculosis* (Xpert MTB/RIF, Cepheid, Sunnyvale, CA, USA), and mycobacterial cultures (BD BACTEC MGIT 960 system, BD, Franklin Lakes, NJ, USA) would be performed by the National Reference TB Laboratory.

### 2.2. Data Collection

Following the enrolment of individuals, the patients’ clinical presentations as well as medical and laboratory records were reviewed. Data, including demographics, microbiological and clinical characteristics, and imaging findings, were collected. A total of 495 individuals were included after fulfilling the inclusion criteria. All the enrolled patients were included in the final analysis. All participants provided written informed consent before enrollment in the study.

### 2.3. Statistical Analysis

Data were analyzed using the Stata software (StataCorp., College Station, TX, USA). Data are presented as the median (IQR) for continuous measures, and n (%) for categorical measures. Pearson’s Chi-squared, Fisher’s exact test, or Wilcoxon rank-sum test were used unless otherwise specified. Sensitivity, negative predictive value, and negative likelihood ratio with a 95% confidence interval were used to evaluate the yield of sputum, sputum bronchoscopy and post-bronchoscopy sputum (PBS) smears, cultures, and nucleic acid amplification testing in the diagnosis of pulmonary TB. Throughout the data analysis, statistical significance was set at *p* < 0.05.

## 3. Results

### 3.1. Demographic and Clinical Characteristics

Over the study period, a total of 495 individuals, with a median age of 31 years (range from 26–38), were included. The majority were male (329, 66.5%), craft and manual workers (428, 86.5%), and had arrived in Qatar within the preceding 6 months (414, 83.6%). South-East Asian patients predominated at 266 (53.7%). The most frequent countries of origin were India (131, 26.5%), the Philippines (123, 24.8%), and Nepal (70, 14.1%). Diabetes mellitus was the most common co-existing medical condition 44 (9%) with median HbA1c 5.5 (5.2–6), and only two patients (0.4%) were HIV-positive. Compared with individuals without confirmed tuberculosis, those with tuberculosis (331, 66.9%) were significantly younger and were more likely to report a history of weight loss and have apical radiological abnormalities, lower baseline hemoglobin, and higher C-reactive protein. Either TST > 5 mm or positive QFT was reported in 263 (79.5%) of the confirmed TB individuals in comparison to 93 (56.7%) without confirmed tuberculosis. Table 1 summarizes the demographic and clinical aspects of the 495 patients involved in this study.

### 3.2. Diagnostic Yield for Individual and Combinations of Tests for Confirmation of Active TB

**Figure 1:**
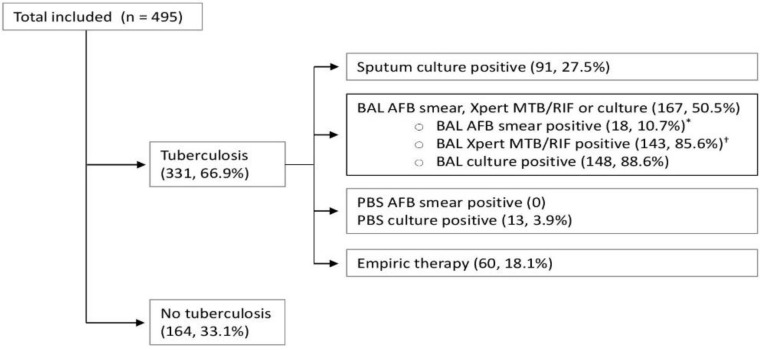
Cumulative diagnostic confirmation of pulmonary tuberculosis for patients who underwent bronchoscopic evaluation as part of their diagnostic work-up. * of which 18 were PCR-positive and 16 were culture-positive. † of which 124 were culture-positive. AFB, acid-fast bacilli; BAL, bronchoalveolar lavage.

Of the 495 suspected TB cases, 331 (66.9%) were diagnosed as pulmonary TB; of these, empiric therapy was initiated in 60 cases (18.1%), Figure 1. The diagnostic yields for the confirmation of active tuberculosis based on individual tests and combinations of tests and samples are summarized in Table 2. AFB smears, NAAT, and cultures for *Mycobacterium tuberculosis* were positive from BAL in 35, 213, and 225 patients, respectively. The sensitivity of any BAL positive tests (AFB smear, Xpert MTB/RIF, or culture) was 74% (69.3–78.7), with a negative predictive value of 65.6% (59.7–71.5) and negative likelihood ratio of 26% (22–31). Among the 331 (66.9%) patients diagnosed with active pulmonary TB, 18 (3.6%) were positive for PBS AFB smears and 131 (26.5%) were positive for PBS *Mycobacterium tuberculosis* culture. The sensitivity of PBS positive results (positive AFB smears, Mycobacterium tuberculosis culture, or both) was 39.9% (34.6–45.2), with a negative predictive value of 45.2% (40.1–50.3) and a negative likelihood ratio of 60% (55–66). The addition of PBS to expectorated sputum and BAL testing allowed microbiological confirmation of tuberculosis in an additional 13 patients (3.9%), resulting in improved sensitivity (from 77.9% to 81.9%), negative predictive value (from 69.2% to 73.2%), and negative likelihood ratio (from 0.22 to 0.18) (Figure 1 and Table 2).

The figures represent numbers and percentages of those with additional positive results and the final disposition as tuberculosis or no tuberculosis.

## 4. Discussion

This was a large prospective study over a two-and-a-half year timeframe that enrolled all eligible patients whose tuberculosis screening included bronchoscopy to collect two PBS samples in a tertiary referral center in the country that basically represented the national profile. Globally, the immediate need to control TB is hindered by negative initial diagnostic tests on expectorated sputum and BAL samples in presumptive active pulmonary tuberculosis cases; hence, there are major implications regarding treatment delay and infectious control [3].

The prevalence of active tuberculosis in the study population is high but is not surprising amongst those who originate from high-prevalence countries and have abnormal chest radiographs [16,17]. Microbiological confirmation of tuberculosis is highly desirable because it allows for diagnostic certainty and susceptibility-guided therapy [1]. This is particularly critical because the management of drug-resistant *Mycobacterium tuberculosis* is challenging, especially when the patient is co-infected with HIV [18]. Therefore, the American Thoracic Society (ATS) and Infectious Diseases Society of America (IDSA) suggest performing post-bronchoscopy sputum examination for AFB smears and *Mycobacterium tuberculosis* culture for presumptive active pulmonary tuberculosis cases who undergo bronchoscopy [9]. However, this is not routinely requested in practice. A likely explanation for this is the limitation of the data in a few studies with considerable uncertainty and variation in the reported estimated diagnostic yields [8,10,11,19]. In this study, PBS testing allowed for additional confirmation and an improved ability to rule out tuberculosis in a high-prevalence population. However, the tuberculosis diagnostic work-up and screening pathway employed in this study require complex logistics and considerable resources. It is utility and cost-effectiveness vary considerably from one setting to another. Notably, we reported that the addition of AFB smears and *Mycobacterium tuberculosis* culture to any expectorated sputum or BAL-positive results increased the sensitivity to 81.9%. This is higher than those previously reported in the literature [8,20,21,22]. This higher rate may be explained by the fact that our institute is a tertiary referral center for presumptive active pulmonary tuberculosis cases; hence, there is a higher possibility of obtaining positive TB results. In addition, some studies collected only one PBS sample [21]. Furthermore, it is not clear whether the detection of AFB in PBS necessitates the same isolation precautions as for patients with open pulmonary tuberculosis. However, undergoing bronchoscopy may mobilize mycobacteria-laden, deep bronchial secretions, resulting in the conversion of smear-negative pulmonary TB to positive and potentially infectious [8]. This can be detrimental and have significant implications for public health and infection control. Thus, we advocate respiratory isolation until confirmation of at least one negative acid-fast smear sample post-bronchoscopy, especially when the pre-test probability of pulmonary TB is high, and the patient is still expectorating.

Notably, symptoms suggestive of tuberculosis were absent in more than 60% of individuals with confirmed tuberculosis in this study. The denial of symptoms could be due to fear of stigmatization or possible denial of residency application [23]. However, a proportion of these patients may have incipient or subclinical tuberculosis [24]. Intensive screening, including PBS testing, can facilitate early detection and treatment initiation for such clinical entities and, hence, avert disease progression and onward transmission. In contrast to our study, other studies have reported higher PBS smear positivity [8,10,11]. However, it is worth mentioning that most of these studies were not designed to investigate the diagnostic yield of BPS samples for TB. Additionally, the small sample size in these studies casts significant doubt on the acceptance of generalization [10,19,20]. Interestingly, the same yield of BAL and PBS cultures has been reported in HIV patients [25,26]. However, only two patients were HIV-positive in our cohort rendering the conclusion invalid. Interestingly, in one of these two patients, the only exclusive confirmation of active pulmonary TB was reported in PBS AFB culture. This could be of importance as multidrug-resistant TB has been reported to be twice as common in HIV-infected patients [27]. Thus, PBS samples can help determine the early diagnosis and drug susceptibility in this group of patients.

To the best of our knowledge, this is the largest study to date to examine the diagnostic yield of PBS as part of a tuberculosis diagnostic work-up [8,12,28]. Our data suggest that PBS sampling can provide a simple and affordable method by which pulmonary TB diagnosis can be further optimized. Moreover, our findings may guide tuberculosis screening and diagnostic pathways in settings where bronchoscopy is readily accessible. However, our findings may have limited generalizability to settings with a high prevalence of tuberculosis. In addition, there is the possibility of selection bias given our center is a referral center for presumptive TB cases and the predomination of South-East Asian patients in our cohort could have confounded the results. Furthermore, in a limited resources center, the increasing cost of further isolation pending the negativity of PBS AFB results may limit the generalizability of the findings.

## 5. Conclusions

Where resources are available, the incorporation of routine PBS examination into tuberculosis work-up and screening procedures can enhance diagnostic yield. In addition, it can provide a simple and accessible additional diagnostic method for the rapid diagnosis of pulmonary TB. By allowing early initiation of treatment and full drug susceptibility testing, appropriate management of potentially drug-resistant TB can be addressed. These could be further steps toward a global strategy to control TB.

## Figures and Tables

**Table 1:** Baseline characteristics of individuals who underwent tuberculosis screening program including bronchoscopy and post-bronchoscopy sputum examination.

	Total (*n* = 495)	Active Tuberculosis (*n* = 331)	No Active Tuberculosis (*n* = 164)	*p* Value
Age—years	31 (26–38)	30 (26–37)	34 (29–41.5)	<0.001
Male sex	329 (66.5%)	226 (68.3%)	103 (62.8%)	0.23
Nationality by WHO region of origin				0.13
African Region	66 (13.3%)	53 (16%)	13 (7.9%)	
Eastern Mediterranean region	36 (7.3%)	23 (7%)	13 (7.9%)	
European region	2 (0.4%)	1 (0.3%)	1 (0.6%)	
Region of America	1 (0.2%)	1 (0.3%)	0	
South-East Asia	266 (53.7%)	171 (51.7%)	95 (57.9%)	
Western Pacific Region	124 (25.1%)	82 (24.8%)	42 (25.6%)	
Craft and manual worker	428 (86.5%)	287 (86.7%)	141 (86%)	0.89
Arrived in Qatar within the preceding 6 months	414 (83.6%)	272 (82.2%)	142 (86.6%)	0.25
Smoker	201 (40.6%)	149 (45%)	52 (31.7%)	0.005
Diabetes	44 (8.9%)	31 (9.4%)	13 (7.9%)	0.74
Hypertension	15 (3%)	8 (2.4%)	7 (4.3%)	0.27
Chronic lung disease	5 (1%)	3 (0.9%)	2 (1.2%)	0.67
Malignancy	2 (0.4%)	1 (0.3%)	1 (0.6%)	0.55
Chronic kidney disease	63 (12.7%)	43 (13%)	20 (12.2%)	0.89
Human immunodeficiency virus infection	2 (0.4%)	2 (0.6%)	0	>0.999
Tuberculosis symptoms	197 (39.8%)	132 (39.9%)	65 (39.6%)	>0.999
Fever	102 (20.6%)	70 (21.2%)	32 (19.5%)	0.72
Weight loss	81 (16.4%)	61 (18.4%)	20 (12.2%)	0.093
Cough	160 (32.3%)	96 (29%)	64 (39%)	0.032
Night sweats	30 (6.1%)	25 (7.6%)	5 (3.1%)	0.07
History of previous tuberculosis	28 (5.7%)	11 (3.3%)	17 (10.4%)	0.003
Family history of tuberculosis	37 (7.5%)	29 (8.76%)	8 (4.88%)	0.15
Bilateral radiological abnormalities	259 (52.3%)	164 (49.6%)	95 (57.9%)	0.086
Apical radiological abnormalities	235 (47.5%)	175 (52.9%)	60 (36.6%)	<0.001
TST > 5 mm *	159 (32.12%)	106 (32.02%)	53 (32.32%)	0.43
QuantiFERON TB Gold positive †	228 (46.1%)	175 (52.9%)	53 (32.32%)	<0.001
TST > 5 mm or QuantiFERON TB Gold positive	356 (71.9%)	263 (79.5%)	93 (56.7%)	<0.001
Hemoglobin counts gm/dL	13.9 (12.9–15.1)	13.8 (12.7–15.1)	14.3 (13.1–15.3)	0.039
Peripheral white cell count (×10^9^/L)	7.7 (6.4–9.4)	7.6 (6.4–9.1)	8 (6.3–9.9)	0.22
Platelet count (10^9^/L)	296 (252–349)	297 (255–349)	291 (241–350)	0.33
C-reactive protein (mg/L)	5.6 (1.8–44.6)	11 (2–50)	3 (0.9–9.4)	0.032
Alanine transaminase (U/L)	18 (13–27)	17.8 (13–26.7)	20.9 (14.3–28)	0.016

Data are presented as median (IQR) for continuous measures, and *n* (%) for categorical measures. Pearson’s Chi squared, Fisher’s exact test, or Wilcoxon rank-sum. * missing for 318 (64.2%) subjects. † missing for 32 (19.5%) subjects. TST, tuberculin skin test.

**Table 2:** Diagnostic yield for the confirmation of active tuberculosis based on individual and combinations of tests and samples.

	Number (%) with Positive Results for *M. tuberculosis*	Sensitivity (95% CI)	Negative Predictive Value (95% CI)	Negative Likelihood Ratio (95% CI)
Expectorate sputum culture	91 (18.4%)	27.5% (22.7–32.3)	40.6% (35.8–45.4)	0.73 (0.68–0.77)
BAL AFB smear	35 (7.1%)	10.6% (7.2–13.9)	35.7% (31.3–40.0)	0.89 (0.86–0.93)
BAL Xpert MTB/RIF	213 (43%)	64.4% (59.2–69.5)	58.2% (52.4–63.9)	0.36 (0.31–0.41)
BAL culture	225 (45.5%)	68.0% (62.9–73.0)	60.7% (54.9–66.6)	0.32 (0.27–0.37)
BAL AFB smear, Xpert MTB/RIF, or culture	245 (49.5%)	74.0% (69.3–78.7)	65.6% (59.7–71.5)	0.26 (0.22–0.31)
Post-bronchoscopy sputum AFB smear	18 (3.6%)	5.4% (3.0–7.9)	34.4% (30.1–38.6)	0.95 (0.92–0.97)
Post-bronchoscopy sputum culture	131 (26.5%)	39.6% (34.3–44.8)	45.1% (39.9–50.2)	0.60 (0.55–0.66)
Post-bronchoscopy sputum AFB smear or culture	132 (26.7%)	39.9% (34.6–45.2)	45.2% (40.1–50.3)	0.60 (0.55–0.66)
Expectorated sputum or BAL AFB smear, culture, or Xpert MTB/RIF	258 (52.1%)	77.9% (73.5–82.4)	69.2% (63.3–75.1)	0.22 (0.18–0.27)
Expectorated sputum, BAL or post-bronchoscopy sputum AFB smear, culture, or Xpert MTB/RIF	271 (54.7%)	81.9% (77.7–86)	73.2% (67.4–79)	0.18 (0.14–0.23)

AFB, acid-fast bacilli; BAL, bronchoalveolar lavage; CI, confidence interval.

## Data Availability

The data that support the findings of this study are available from the corresponding author, (WG), upon reasonable request.
